# Racial and Ethnic Disparities in Sustained Viral Suppression and Transmission Risk Potential Among Persons Receiving HIV Care — United States, 2014

**DOI:** 10.15585/mmwr.mm6704a2

**Published:** 2018-02-02

**Authors:** Nicole Crepaz, Xueyuan Dong, Xiao Wang, Angela L. Hernandez, H. Irene Hall

**Affiliations:** 1 Division of HIV/AIDS Prevention, National Center for HIV, Viral Hepatitis, STD and TB Prevention, CDC.

Non-Hispanic blacks/African Americans (blacks) represent 12% of the U.S. population.* However, in 2014 an estimated 43% (471,500) of persons living with diagnosed and undiagnosed human immunodeficiency virus (HIV) infection were blacks (*1*). In 2016, blacks accounted for 44% of all new HIV diagnoses (*2*). Although antiretroviral therapy (ART) prescriptions among persons in HIV care increased overall from 89% in 2009 to 94% in 2013, fewer blacks than Hispanics or Latinos (Hispanics) and non-Hispanic whites (whites) were on ART and had a suppressed viral load (<200 HIV RNA copies/mL) in their most recent viral load test result (*3*). Blacks also might be less likely to have sustained viral suppression over time and to experience longer periods with viral loads >1,500 HIV RNA copies/mL, a level that increases the risk for transmitting HIV (*4*–*7*). National HIV Surveillance System (NHSS) data are among those used to monitor progress toward reaching the national goal of reducing health disparities. CDC analyzed NHSS data to describe sustained viral suppression and transmission risk potential by race/ethnicity. Among 651,811 persons with HIV infection diagnosed through 2013 and who were alive through 2014 in 38 jurisdictions with complete laboratory reporting, a lower percentage of blacks had sustained viral suppression (40.8%), than had Hispanics (50.1%) and whites (56.3%). Among persons who were in care (i.e., had at least one viral load test in 2014) and had not achieved sustained viral suppression in 2014, blacks experienced longer periods (52.1% of the 12-month period) with viral loads >1,500 copies/mL, than did Hispanics (47.2%) and white (40.8%). Blacks aged 13–24 years had the lowest prevalence of sustained viral suppression, a circumstance that might increase transmission risk potential. Strengthening interventions that improve access to ART, promote adherence, and address barriers to clinical care and supportive services for all persons with diagnosed HIV infection is important for achieving the national goal of reducing health disparities.

All states, the District of Columbia (DC), and U.S. territories report cases of HIV infection and associated demographic and clinical information to NHSS. CDC analyzed data from NHSS reported through June 2017 from 37 states and DC with complete laboratory reporting. These jurisdictions accounted for 71.9% of persons living with diagnosed HIV infection at the end of 2014 in the United States. This analysis includes persons aged ≥13 years who received a diagnosis of HIV infection by December 31, 2013, most recently resided in one of the 38 jurisdictions, and were alive at the end of 2014. For persons who had two or more viral load tests in 2014, sustained viral suppression was defined as viral load test results of <200 copies of HIV RNA/mL for all tests in 2014. For persons who had only one viral load test in 2014, sustained viral suppression was defined as a viral load test result of <200 copies/mL for the 2014 test and also for the last viral load test in 2013. Both groups were included in the numerator. Persons with partial viral suppression in 2014 (i.e., some, but not all viral load test results <200 copies/mL) were excluded from the numerator but included in the denominator. Persons with no viral load tests in 2014 were presumed not to be suppressed and were excluded from the numerator. The numbers and percentages of persons with sustained, partial, and no viral suppression were calculated. All persons living with diagnosed HIV at the end of 2014 were included in the denominator for determining the percentage with sustained viral suppression.

HIV transmission potential was estimated among persons in care who did not achieve sustained viral suppression and was defined as the number of days that a person’s viral load was >1,500 copies/mL. The estimated number of days with viral load >1,500 copies/mL was calculated for each person and then was averaged across the analytic cohort (*5*,*6*). Persons with no viral load test in 2014 were considered to be not in care and were not included when calculating transmission potential. Sustained viral suppression and transmission risk potential were assessed by sex, age, and transmission category, stratified by race/ethnicity. Data were adjusted using multiple imputation to account for 17.9% missing HIV transmission categories (*8*).

In the 38 jurisdictions, 651,811 persons with HIV infection diagnosed through 2013 were alive at the end of 2014, including 263,588 (40.4%) blacks, 199,700 (30.6%) whites, 149,117 (22.9%) Hispanics, and 39,406 (6.1%) other racial/ethnic groups (data for other racial/ethnic groups not shown). The median number of viral load tests in 2014 was two, with 356,223 (54.7%) persons having two or more tests, 95,926 (14.7%) having one test, and 199,662 (30.6%) having no test in 2014. The percentage of persons without a viral load test in 2014 was 33.9% among blacks, 29.9% among Hispanics, and 28.2% among whites.

Among all persons living with diagnosed HIV infection in the 38 jurisdictions, 48.4% had sustained viral suppression in 2014. A lower proportion of blacks had sustained viral suppression (40.8%), than did Hispanics (50.1%) and whites (56.3%). Across the sex, age, and transmission category subgroups, the proportion of blacks with sustained viral suppression was lower than that of Hispanics and whites (Table 1). Blacks aged 13–24 years had the lowest prevalence of sustained viral suppression (29.2%).

**TABLE 1:** Sustained viral suppression* among persons aged >13 years with human immunodeficiency virus (HIV) infection diagnosed through 2013 who were alive at the end of 2014, by race/ethnicity and selected characteristics^†^ — National HIV Surveillance System, 37 states and the District of Columbia,^§,¶^ 2014

Characteristic	Racial/Ethnic group, No. (%)							
	All groups**		Black		Hispanic/Latino		White	
	Total	Sustained viral suppression	Total	Sustained viral suppression	Total	Sustained viral suppression	Total	Sustained viral suppression
**Total**	**651,811 (100.0)**	**315,390 (48.4)**	**263,588 (100.0)**	**107,438 (40.8)**	**149,117 (100.0)**	**74,721 (50.1)**	**199,700 (100.0)**	**112,413 (56.3)**
**Sex**								
Male	500,057 (76.7)	246,950 (49.4)	175,170 (66.5)	70,398 (40.2)	118,621 (79.5)	59,235 (49.9)	175,690 (88.0)	100,820 (57.4)
Female	151,754 (23.3)	68,440 (45.1)	88,418 (33.5)	37,040 (41.9)	30,496 (20.5)	15,486 (50.8)	24,010 (12.0)	11,593 (48.3)
**Age group at diagnosis (yrs)**								
13–24	27,825 (4.3)	9,380 (33.7)	16,328 (6.2)	4,769 (29.2)	6,086 (4.1)	2,470 (40.6)	3,544 (1.8)	1,461 (41.2)
25–34	95,460 (14.6)	38,714 (40.6)	45,207 (17.2)	15,297 (33.8)	24,744 (16.6)	11,022 (44.5)	19,058 (9.5)	9,389 (49.3)
35–44	144,068 (22.1)	66,250 (46.0)	58,074 (22.0)	22,885 (39.4)	38,286 (25.7)	18,469 (48.2)	37,869 (19)	19,819 (52.3)
45–54	223,990 (34.4)	114,726 (51.2)	83,043 (31.5)	36,480 (43.9)	49,524 (33.2)	25,893 (52.3)	78,538 (39.3)	45,208 (57.6)
≥55	160,468 (24.6)	86,320 (53.8)	60,936 (23.1)	28,007 (46.0)	30,477 (20.4)	16,867 (55.3)	60,691 (30.4)	36,536 (60.2)
**Transmission category**								
**Male**								
Male-to-male sexual contact	357,258 (54.8)	185,535 (51.9)	107,769 (40.9)	44,248 (41.1)	82,991 (55.7)	43,790 (52.8)	144,148 (72.2)	85,041 (59.0)
Injection drug use	54,485 (8.4)	21,559 (39.6)	26,708 (10.1)	9,815 (36.7)	15,971 (10.7)	6,346 (39.7)	9,338 (4.7)	4,201 (45.0)
Male-to-male sexual contact and injection drug use	39,225 (6.0)	18,530 (47.2)	11,747 (4.5)	4,796 (40.8)	8,724 (5.9)	4,030 (46.2)	15,640 (7.8)	8,192 (52.4)
Heterosexual contact	43,859 (6.7)	19,313 (44.0)	26,749 (10.1)	10,886 (40.7)	9,674 (6.5)	4,566 (47.2)	5,156 (2.6)	2,687 (52.1)
**Female**								
Heterosexual contact	110,865 (17.0)	51,331 (46.3)	67,415 (25.6)	28,851 (42.8)	21,754 (14.6)	11,563 (53.2)	15,459 (7.7)	7,774 (50.3)
Injection drug use	36,267 (5.6)	15,472 (42.7)	18,556 (7.0)	7,426 (40.0)	7,580 (5.1)	3,481 (45.9)	7,846 (3.9)	3,511 (44.7)
**Other**	9,853 (1.5)	3,651 (37.1)	4,644 (1.8)	1,417 (30.5)	2,424 (1.6)	945 (39.0)	2,112 (1.1)	1,007 (47.7)

*Defined as all viral load test results <200 HIV RNA copies/mL in 2014. The cutoff value of <200 HIV RNA copies/mL was based on the U.S. Department of Health and Human Services recommended definition of virologic failure (i.e., failure of antiretroviral therapy to suppress a viral load to <200 copies/mL).

^†^ Because the column totals were calculated independently of the corresponding values for each population group, the individual values might not sum to the totals.

^§^ The 38 jurisdictions were Alabama, Alaska, California, Colorado, Connecticut, Delaware, District of Columbia, Georgia, Hawaii, Illinois, Indiana, Iowa, Louisiana, Maine, Maryland, Massachusetts, Michigan, Minnesota, Mississippi, Missouri, Montana, Nebraska, New Hampshire, New Mexico, New York, North Dakota, Oregon, Rhode Island, South Carolina, South Dakota, Tennessee, Texas, Utah, Virginia, Washington, West Virginia, Wisconsin, and Wyoming.

^¶^ Percentages for the totals are column percentages; viral suppression percentages are row percentages.

** Includes all racial/ethnic groups (blacks, Hispanics/Latinos, whites, and others).

Among 136,759 persons who were in care in 2014, but did not achieve sustained viral suppression, 89,245 (65%) had at least one viral load test result of >1,500 copies/mL in 2014. Overall, the mean number of days with a viral load >1,500 copies/mL was 176 (48.3% of the 12-month period). The mean number of days with a viral load >1,500 copies/mL was higher among blacks (190 days, 52.1% of the 12-month period) than among Hispanics (172 days, 47.2%) and whites (149 days, 40.8%) (Table 2). Across all sex, age, and transmission category subgroups, blacks experienced a longer percentage of time during 2014 with viral loads >1,500 copies/mL than did Hispanics and whites (Figure). Blacks aged 13–24 years experienced the highest percentage of time with viral load >1,500 copies/mL (216 days, 59% of the 12-month period).

**TABLE 2:** Transmission risk potential* among persons aged ≥13 years with human immunodeficiency virus (HIV) infection diagnosed through 2013 who were alive at the end of 2014, by race/ethnicity and selected characteristics — National HIV Surveillance System, 37 states and the District of Columbia,^†^ 2014

Characteristic	Mean no. of days during 2014 with viral load >1,500 copies/mL			
	Overall^§^	Black	Hispanic/Latino	White
	(n = 136,759)	(n = 66,677)	(n = 29,684)	(n = 31,033)
**Total**	**176**	**190**	**172**	**149**
**Sex**				
Male	174	191	171	145
Female	184	188	175	173
**Age group at diagnosis (yrs)**				
13–24	211	216	209	192
25–34	204	212	198	187
35–44	186	197	179	170
45–54	164	179	160	142
≥55	136	156	131	103
**Transmission category**				
**Male**				
Male-to-male sexual contact	171	195	170	138
Injection drug use	172	178	166	159
Male-to-male sexual contact and injection drug use	183	189	182	176
Heterosexual contact	178	187	167	142
**Female**				
Heterosexual contact	182	188	171	165
Injection drug use	184	185	179	186
**Other**	199	209	206	151

* Defined as number of days with a viral load above 1,500 HIV RNA copies/mL during a 12-month period in 2014. Risk for transmission increases when viral load >1500 copies/mL.

^†^ The 38 jurisdictions were Alabama, Alaska, California, Colorado, Connecticut, Delaware, District of Columbia, Georgia, Hawaii, Illinois, Indiana, Iowa, Louisiana, Maine, Maryland, Massachusetts, Michigan, Minnesota, Mississippi, Missouri, Montana, Nebraska, New Hampshire, New Mexico, New York, North Dakota, Oregon, Rhode Island, South Carolina, South Dakota, Tennessee, Texas, Utah, Virginia, Washington, West Virginia, Wisconsin, and Wyoming.

^§^ Includes all racial/ethnic groups (blacks, Hispanics/Latinos, whites, and others).

**FIGURE:**
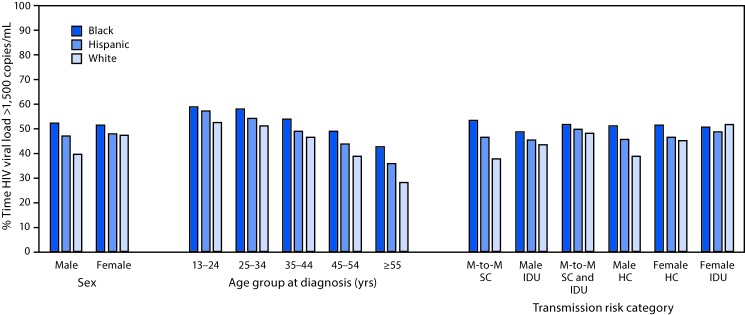
Percentage of time during 2014 when human immunodeficiency virus (HIV) viral load was >1,500 copies/mL among persons aged ≥13 years with HIV infection diagnosed through 2013 who were alive at the end of 2014, by race/ethnicity, sex, age group, and transmission risk category — 37 states and the District of Columbia, 2014 **Abbreviations:** HC = heterosexual contact; IDU = injection drug use; M-to-M = male-to-male SC = sexual contact.

## Discussion

Viral suppression is essential to maintaining the health of persons living with HIV infection and reducing the likelihood of HIV transmission. National treatment guidelines recommend that all persons with diagnosed HIV infection, regardless of their viral load or CD4 level, take ART to achieve viral suppression.^†^ However, only 40.8% of blacks living with diagnosed HIV infection in 38 jurisdictions with complete laboratory reporting had sustained viral suppression in 2014, a percentage lower than that among Hispanics (50.1%) and whites (56.3%). The remaining 59.2% of blacks included 25.3% who were in care but did not have sustained viral suppression in 2014 (i.e., partial suppression or not suppressed) and 33.9% with no viral load tests in 2014. The latter is an indication of not receiving adequate HIV care and presumably not having suppressed viral load. Among those in care, blacks experienced a longer period (i.e., half of the time during the 12-month period) with a viral load >1,500 copies/mL, a circumstance which can adversely affect health outcomes and pose a risk for further transmission. Although prescription of ART increased among blacks who received HIV clinical care from 2009 to 2013, fewer blacks received an ART prescription (92.9%) than did Hispanics (95.2%) and whites (95.2%) (*3*). These findings highlight areas for improvement in care retention and offering of ART to all persons with HIV infection according to the national treatment guidelines.

The racial/ethnic differences in sustained viral suppression were present across all sex, age, and transmission categories, and the lowest prevalence of sustained viral suppression was found among blacks aged 13–24 years. Lower viral suppression, combined with the higher prevalences of HIV among blacks compared with other racial/ethnic groups, could lead to a higher HIV transmission risk potential. Barriers such as lack of health insurance, limited access to health services, stigma, health literacy, and lack of trust in providers and the care system might be contributing to these disparities (*9*). Addressing barriers to care and treatment is important to improving the health of persons living with HIV and reducing disparities.

The findings in this report are subject to at least three limitations. First, analyses were limited to 38 jurisdictions with complete reporting of all levels of CD4 and viral load test results; these jurisdictions might not be representative of all persons living with diagnosed HIV infection in the United States. Second, persons might have moved out of a jurisdiction after their latest address was recorded in the 38 jurisdictions, and this migration might contribute to missing viral load records. Finally, 30.6% of 651,811 persons living with a diagnosis of HIV did not have any viral load test in 2014 and were not included in the analysis for transmission risk potential. Many of these persons might not have had a suppressed viral load and might have experienced longer periods with viral loads >1,500 copies/mL. The transmission risk potential for this group is likely to be high, but cannot be determined because of unavailability of viral load data.

Addressing ongoing racial/ethnic disparities in sustained viral suppression is important to efforts to reduce HIV infections in the United States. CDC is pursuing a high-impact prevention approach that combines scientifically proven, cost-effective, and scalable interventions, including expanding HIV testing and increasing treatment adherence (*10*) to reduce HIV infections and increase the effectiveness of HIV prevention and care activities. CDC supports projects that aim to reduce undiagnosed infections, improve engagement in care, and increase sustained viral suppression across all racial/ethnic groups. To reach the national goal of reducing health disparities, tailored strategies that address barriers to achieving and sustaining viral suppression among blacks, especially those aged 13–24 years, are needed. Continued collaboration among health care providers, community-based organizations, and state and local health departments might strengthen programs that address those barriers.

**Summary**

**What is already known about this topic?**

African Americans/Blacks (blacks) accounted for a disproportionally high percentage of persons living with diagnosed human immunodeficiency virus (HIV) infection in 2014. Between 2009 and 2013, antiretroviral therapy prescriptions have increased more among blacks who received HIV clinical care compared with Hispanics and whites. However, fewer blacks received antiretroviral therapy prescriptions compared with other racial/ethnic groups, and more blacks did not have a suppressed viral load.

**What is added by this report?**

In 2014, fewer blacks living with diagnosed HIV infection had sustained viral suppression (all viral load test results in 2014 <200 HIV RNA copies/mL) compared with Hispanics and whites. Among those who were in care and did not achieve sustained viral suppression, blacks had viral loads >1,500 copies/mL for approximately half of the 12-month period in 2014; this circumstance can adversely affect their health outcomes and pose a risk for further transmission. Blacks aged 13–24 years had the lowest prevalence of sustained viral suppression.

**What are the implication for public health practice?**

Collaboration among health care providers, community-based organizations, and state and local health departments to strengthen programs that address barriers to HIV care, antiretroviral therapy prescription, medication adherence, and sustained viral suppression among blacks, especially blacks aged 13–24 years, could be beneficial in eliminating racial/ethnic disparities.

## Footnotes

* https://www.census.gov/programs-surveys/popest/data/data-sets.2016.html.

† https://aidsinfo.nih.gov/ContentFiles/AdultandAdolescentGL.pdf.
